# Neural network system for analyzing statistical factors of patients for predicting the survival of dental implants

**DOI:** 10.3389/fninf.2022.1067040

**Published:** 2022-12-07

**Authors:** Pavel Alekseevich Lyakhov, Alexander Alexandrovich Dolgalev, Ulyana Alekseevna Lyakhova, Alexandr Alexandrovich Muraev, Kirill Evgenievich Zolotayev, Dmitry Yurievich Semerikov

**Affiliations:** ^1^North-Caucasus Federal University, Stavropol, Russia; ^2^Stavropol State Medical University of the Ministry of Health of the Russian Federation, Stavropol, Russia; ^3^Peoples’ Friendship University of Russia, Moscow, Russia; ^4^Limited Liability Company “Valentina Dental Clinic”, Nyagan, Russia

**Keywords:** big data, digital data processing, dentistry, dental implantation, survival, data mining, artificial neural network, health information technology

## Abstract

Implants are now the standard method of replacing missing or damaged teeth. Despite the improving technologies for the manufacture of implants and the introduction of new protocols for diagnosing, planning, and performing implant placement operations, the percentage of complications in the early postoperative period remains quite high. In this regard, there is a need to develop new methods for preliminary assessment of the patient’s condition to predict the success of single implant survival. The intensive development of artificial intelligence technologies and the increase in the amount of digital information that is available for analysis make it relevant to develop systems based on neural networks for auxiliary diagnostics and forecasting. Systems based on artificial intelligence in the field of dental implantology can become one of the methods for forming a second opinion based on mathematical decision making and forecasting. The actual clinical evaluation of a particular case and further treatment are carried out by the dentist, and AI-based systems can become an integral part of additional diagnostics. The article proposes an artificial intelligence system for analyzing various patient statistics to predict the success of single implant survival. As the topology of the neural network, the most optimal linear neural network architectures were developed. The one-hot encoding method was used as a preprocessing method for statistical data. The novelty of the proposed system lies in the developed optimal neural network architecture designed to recognize the collected and digitized database of various patient factors based on the description of the case histories. The accuracy of recognition of statistical factors of patients for predicting the success of single implants in the proposed system was 94.48%. The proposed neural network system makes it possible to achieve higher recognition accuracy than similar neural network prediction systems due to the analysis of a large number of statistical factors of patients. The use of the proposed system based on artificial intelligence will allow the implantologist to pay attention to the insignificant factors affecting the quality of the installation and the further survival of the implant, and reduce the percentage of complications at all stages of treatment. However, the developed system is not a medical device and cannot independently diagnose patients. At this point, the neural network system for analyzing the statistical factors of patients can predict a positive or negative outcome of a single dental implant operation and cannot be used as a full-fledged tool for supporting medical decision-making.

## Introduction

To date, artificial dental implants are the standard method for replacing damaged or missing teeth. More than a million implants are surgically placed in patients every year (le Guéhennec et al., 2007). This raises the problem of qualitative selection of the correct location of the implant, the proper diameter and length, as well as the material of the prosthesis to minimize the risk of rejection (Cobo-Vázquez et al., 2018). The percentage of biological complications that lead to implant rejection in the first year after implant placement is quite high and ranges from 2 to 5%. The mean 10-year survival rate for a single implant is 96.4% (Howe et al., 2019). In the subsequent period of functional load on the implant structure, technical complications come to the fore, which consist of a fracture of the superstructure or the implant itself. The percentage of technical complications reaches 15% in the first 3–5 years of operation of the structure (Roos-Jansåker et al., 2006). Up to 80% of all complications are associated with errors in diagnosis and treatment planning at the initial stage, which makes research on improving implant survival rates and prolonging the life of the prosthetic structure relevant for modern dentistry and maxillofacial surgery (den Hartog et al., 2008).

Today, medicine is considered one of the strategic and promising areas for the effective implementation of artificial intelligence (Alegre-Cortés et al., 2018). Neural network technologies have the potential to discover relationships and patterns in big data (Kim et al., 2018) and may allow computers to perform assisted disease diagnosis and prediction, as well as suggest further treatment options (Chan et al., 2018). The increase in the volume of information in the field of dental implantology, as well as the need to extract knowledge from this information, is the main reason for the development and use of data analysis systems based on artificial intelligence (Hashem et al., 2020; Alharbi and Almutiq, 2022). Artificial intelligence in dentistry is a method for generating an informed second opinion that is based on mathematical decision making and prediction (Bernauer et al., 2021; Choudhury et al., 2022). Machine learning is generally not intended to replace the dental professional (Carrillo-Perez et al., 2022). Neural network technologies are used in such areas as the analysis of dental radiographs (Lee et al., 2020), the prediction of the need for oral treatment in children (Wang et al., 2020), the classification of dental deposits and treatment planning for orthognathic surgery (Choi et al., 2019), and the auxiliary diagnosis of caries (Mansour et al., 2019).

## Related work

Currently, the use of artificial intelligence technologies in clinical decision support systems in the field of dentistry is relevant (Khanna, 2010; Jiang et al., 2017). Expert systems based on neural networks can only be trained on clinical data and can be used in cases where “rule-based” decision making is not possible. This happens in many clinical situations, so intelligent systems can become an important decision-making tool in dentistry. The study Kim et al. (2009) proposed a toothache prediction model based on neural network algorithms. By analyzing patient statistics, the algorithm was trained to look for relationships between toothache and brushing frequency, brushing time (before meals or after meals), brushing learning experience, flossing, toothbrush replacement frequency, and other factors including diet. and exercise. A three-layer perceptron architecture with 131 input neurons, 6 hidden layers and an output layer was used as a neural network topology. As a result, a predictive model for the development of toothache was obtained with an accuracy of about 80%. The model identified proper nutrition, oral hygiene, and stress reduction as the most important factors in preventing toothache.

In Xie et al. (2010), an expert decision-making system based on artificial intelligence technologies was proposed to determine the need for extraction before orthodontic treatment. The proposed neural network topology had 23 neurons in the input layer and 1 neuron in the output layer. The training of the neural network system was carried out using the backpropagation algorithm. The simulation results showed that the proposed artificial neural network in this study can correctly judge the need for extraction before orthodontic treatment of patients with malocclusion aged 11–15 years with an accuracy of up to 80%.

In the study Miladinović et al. (2010), artificial intelligence technologies were used to analyze the indirect cause of tooth extraction based on the processing of a large volume of electronic medical records. As the topology of the neural network, a linear classifier consisting of two layers was proposed. The immediate reasons for extraction in 5,257 cases were dental caries (43.8%), periodontal disease (37.2%), fractures (6.8%), prostheses (4.3%), retention (3.1%), orthodontics (2.7%), milk teeth (0.3%). As a result of the data mining of electronic medical records of selected subjects using the proposed algorithm, it was confirmed that the number of extracted teeth was statistically influenced by gender, age and occupation. This algorithm made it possible to identify factors influencing the need for tooth extraction based on cause-and-effect relationships.

In Käkilehto et al. (2009), big data mining was performed to determine whether differences in materials during restoration are a determining factor in service life. As a result, it was found that the average service life of amalgam occlusal restorations was 16.8 years in the 1960 patient group, 13.6 years in the 1970 patient group, and 7.9 years in the 1980 patient group of glass ionomer and composite on the occlusal surface were 4.9 years in the 1970 patient group and 7.3 years in the 1980 patient group. The study clearly shows that undocumented information in large volumes over many years can be extracted and analyzed using data mining.

In the field of dental implantology, there is also an active introduction of decision support systems based on artificial neural networks. In Oliveira et al. (2005), the success of implant treatment was studied using several data mining algorithms. Algorithms based on neural networks, support vector machines and K-nearest neighbors have been proposed. The simulation showed that the accuracy of the proposed algorithms in diagnosing the success of implantation was 75.5, 75.9, and 75.9%, respectively. Statistical factors affecting implant survival in this study were age, gender, implant position, implant type, surgical technique, smoking, and previous illness. For a binary assessment of the probability of success or failure in the survival of a dental implant, a model based on artificial neural networks was proposed in Braga et al. (2012). As a result of training and testing various models, the largest value of the AUC estimation parameter was 0.789 and showed that in 78.9% of the prediction cases, the proposed model matches the test data. In the work (Sadighpour et al., 2014), based on artificial intelligence, a clinical decision support system for the installation of maxillary implants for a patient with a completely edentulous upper jaw is presented. Case histories of 47 patients were included in the study. An architecture consisting of one input layer, one hidden layer, and one output layer was used as the topology of the neural network. The accuracy of the decision support system network was 83.3%.

In all the works reviewed, it was proved that decision support systems based on artificial intelligence, which was trained on the data obtained as a result of dentists’ decisions, can be used as auxiliary tools. Studies show the relevance and prospects of using neural network technologies in modern dentistry for auxiliary diagnostics and prognosis by extracting meaningful information from a large number of medical records to create expert systems that will help dentists make decisions (Roongruangsilp and Khongkhunthian, 2021). Data mining and expert systems are based on a large amount of previous data regarding dental diagnosis, treatment and professional judgment, which is converted into text and numbers (Lee et al., 2012). The actual clinical evaluation and treatment are carried out by the dentist all the time and artificial intelligence can become an integral part of assisted diagnosis and further treatment (Pethani, 2021).

Despite significant progress in the implementation of artificial intelligence technologies in the field of dental implantology, the development of neural network systems for supporting medical decision-making of varying complexity is relevant for achieving higher accuracy in predicting the success of implant survival (Revilla-León et al., 2021). The main hypothesis of the article is a potential increase in the survival rate of dental implants due to the development and implementation of a system for predicting the success rate of single implants based on artificial intelligence as an additional auxiliary tool. The purpose of this study is to develop and model a neural network system for analyzing various statistical factors of patients to assess the success of single implant survival. The proposed neural network system makes it possible to achieve higher recognition accuracy than similar neural network systems by collecting and analyzing a large number of digitized statistical factors of patients and selecting the optimal neural network architecture for analyzing the obtained factors. The use of the proposed system based on artificial intelligence should help the implantologist to pay attention to the most significant factors affecting the quality of the installation and further survival of the implant, and reduce the percentage of complications at all stages of treatment.

The rest of the work is structured as follows. Section “Related work” is divided into several subsections. In subsection 3.1. A description of the collected and digitized case histories of patients who underwent surgical osseointegration is presented. In subsection 3.2. A pre-processing method by encoding the collected statistical factors is described. In subsection 3.3. A neural network architecture for the analysis of statistical factors based on a multilayer linear perceptron is presented. Section “Materials and methods” presents a practical simulation of the proposed system for analyzing patient statistical factors to assess the success of single implant survival based on artificial intelligence. Section “Results” discusses the obtained results and their comparison with known systems based on artificial intelligence for predicting the success rate of dental implants. In conclusion, the results of the work are summarized.

## Materials and methods

**FIGURE 1:**
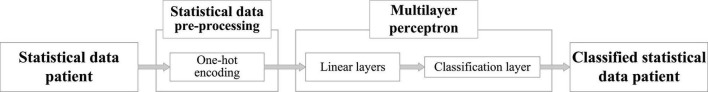
Scheme of the proposed neural network system for predicting the survival rate of single implants.

The paper proposes a system for predicting the survival rate of single implants based on artificial intelligence. The proposed neural network system analyzes the statistical data of patients represented by various factors influencing the success of single implants. The scheme of the proposed neural network system for classifying cases of implantation in surgical dental treatment is shown in Figure 1.

Patient statistics are pre-processed with coding to create a feature vector. The proposed neural network system consists of a linear multilayer perceptron. The resulting feature vector obtained after passing through all the layers of the perceptron is fed to the *softmax* output layer. The output signal of the proposed neural network system for predicting the success of single implant survival is the percentage for 2 diagnostic categories.

Artificial intelligence-based systems in the field of dental implantology are especially useful for processing and analyzing large amounts of data to classify results, as well as for handling repetitive workflows. AI algorithms enable evidence-based dental decision support, especially for less experienced practitioners, and facilitate the analysis of individual patient cases.

The proposed neural network system makes it possible to achieve higher recognition accuracy than similar neural network prediction systems by analyzing a large number of digitized statistical factors of patients. The use of the developed system based on artificial intelligence as an additional auxiliary tool will allow the dentist to pay attention to the factors that affect the quality of the installation and further survival of the implant and reduce the percentage of complications at all stages of treatment.

### Statistical data for modeling a neural network system for predicting implant survival

To date, in the field of dentistry, there is an increase in the volume of digital information due to the accumulation of the results of laboratory and instrumental studies, data from electronic medical records (Andreu-Perez et al., 2015). Patients’ medical statistics are structured data that describe the characteristics of research subjects and include parameters such as gender, age, race, predisposition to various diseases, chronic diseases, bad habits, etc (Shugaa-Addin et al., 2016). The analysis of such statistical information of patients using neural network technologies facilitates the search for links between the objects of study and the result of diagnosis and treatment (Goh, 2020). Digitization of patient records is an important task in the field of introducing information technologies into medicine, since the formation of electronic databases of medical information and their further processing can be used to build intelligent diagnostic systems and decision support for specialists, doctors, and clinicians (Hardin and Chhieng, 2007).

To develop and conduct further modeling of a system for predicting the success of a single implant survival based on artificial intelligence, a database of clinical cases of patient implantation was collected based on a description of the case histories. The primary experimental sample consisted of a description of 1,646 patient histories, taking into account 112 factors affecting osseointegration, as well as the technical characteristics of the implant, orthopedic and surgical protocols for the operation, and facts of identified complications at each stage. Each case of surgical dental treatment was tracked for the fact of implant rejection and recorded in the collected database. A distinctive feature of the sample base of clinical cases is the collection of data from retrospective and prospective studies obtained according to a single protocol from dental implantation centers located in different geographical locations. The scatter in the geographical location of patients who underwent implant treatment made it possible to collect the most representative sample of clinical cases.

**FIGURE 2:**
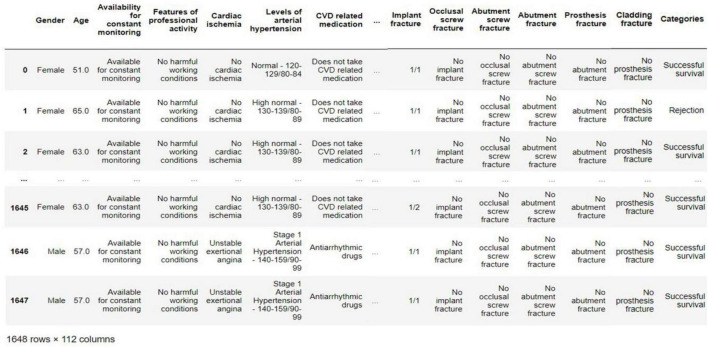
An example of the collected database of clinical cases of implantation of patients.

All collected clinical cases were digitized in the form of a data table with values for each factor from the processed case history. All factors were divided into such groups as: the general somatic condition of the patient, the state of the dentition, the state of the perceiving bed, the characteristics of the implant, surgical and orthopedic protocols, and biological complications at each stage. When forming the primary sample, physicians and specialists in the field of dental implantology conducted a factor analysis to identify the most significant factors in the first approximation. Figure 2 shows an example of the collected and digitized database of clinical cases of implantation of patients based on the description of case histories.

### Pre-processing of patient statistics

Pre-processing of the collected data of an experimental sample of clinical cases based on the description of patient histories is the transformation of statistical data into the format required by the selected data mining method. Since the developed system for predicting the success of implant survival is a fully connected neural network, the collected database must be converted as a feature vector. For each case of patient implantation, an appropriate metadata information vector is created from the experimental sample, which depends on the amount and type of statistical information.

One method of creating an input information vector is to convert all the variables of each case using one-hot encoding (Karthiga et al., 2021). This coding method is the most common and can sometimes outperform more complex statistical information coding systems (Potdar et al., 2017). When processing data using one-hot encoding, variables with multiple possible values are converted into a new set of numeric positional vectors, all elements of which are zero except for the position of the variable’s value in the list of all possible values (El Affendi and Al Rajhi, 2018). For example, a categorical variable indicating the presence of periodontal disease in a patient can take on values such as absence of disease, localized gingivitis, localized periodontitis, generalized gingivitis, generalized periodontitis and, when processed using the one-hot encoding method, will be replaced by five dummy variables indicating the presence of a possible value of the variable. The one-hot encoding method assumes that all categories and factors are independent and allows you to learn a separate parameter for each case without sharing parameters (Seger, 2018). When studying the statistical factors of patients that affect the success of single implants, the independence of each individual variable during neural network analysis is an important criterion when choosing a method for encoding input data.

Let the collected database of statistical data of patients *D* may include factors influencing osseointegration *D* = {*D*_1_,*D*_2_,*D*_3_,…,*D*_*k*_}, while *D*_*k*_ ∈ *d*_*k*_, where *d_k_* is a pointer to a specific factor of the patient. If the variable *d_k_* is a pointer to the factor of allergic reactions in the patient, then *D*_1_ = {*no allergy*; *drug allergy*; *household allergy*; *food allergy*}. For each set of *D_k_*, which is one of the factors of the patient, its power is calculated:


(1) $$\mu_{k}=|D_{k}|,$$

For preliminary processing of the base of factors affecting implant survival, a vector of size features is generated:


(2) $$\vec{d}=\sum_{k}\mu_{k},$$

where μ_*k*_ is the *D_k_* set cardinality, which is one of the patient factors. The first coordinate of the feature vector of the statistics data $\vec{d}$ of dimension μ_1_ encodes the statistical factor *d_1_*. The next coordinate of the μ_2_ the dimension will encode the *d_2_* statistical data, and so on.

**FIGURE 3:**
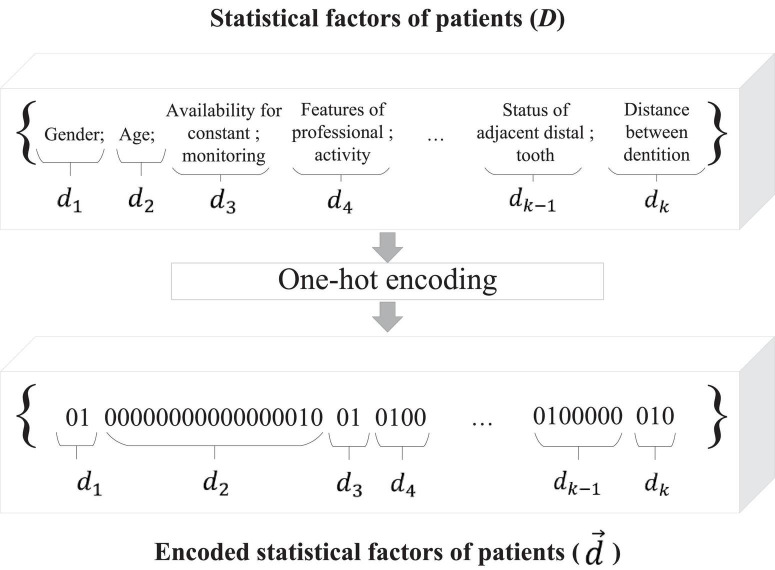
Scheme of pre-processing of statistical factors of patients influencing the success of implant survival.

The one-hot encoding method is used to transform the statistical data in such a way that the set of *d*_*k*_ ∈ *D*_*k*_ is ordered in an arbitrary fixed way for all considered factors. The binary code $\underset{\mu_{k}}{\underbrace{100\,\ldots \,0}}$is reserved for the first element *d_1_* of the set *D_k_*. For the second element *d_2_* of the set *D_k_*, the binary code $\underset{\mu_{k}}{\underbrace{010\,\ldots \,0}}$is reserved, and so on. The scheme of preliminary processing of statistical factors of patients by the one-hot encoding method is shown in Figure 3.

### Neural network system for predicting the success of implant survival

Artificial neural networks are the backbone of most deep learning algorithms due to their flexibility and great learning ability (Lee and Choeh, 2014). Artificial neural networks are a system of artificial neurons that are interconnected and interact (Lakhotia and Bresson, 2018). The application of artificial intelligence algorithms in dentistry is a promising area of research because it allows the identification of certain patterns from large databases and signals (Sabzekar et al., 2021). Artificial intelligence in dentistry is a method of creating a second cogent opinion, which is based on mathematical decision making and prediction (Park and Park, 2018; Bernauer et al., 2021).

The main properties of a neural network are set by the choice of its topology (Muh Ibnu Choldun et al., 2019). The organization of neurons and their connections into a certain structure has a significant impact on the computational capabilities of a neural network (Ru et al., 2022). The most common type of artificial neural networks is the feedforward multilayer perceptron, in which the connections between neurons do not form a loop (Lyu et al., 2022). The neurons perform the summation of the received weighted input data *v* and the bias value *p*, forming a synaptic input. As a result of training on a dataset with known labels, the neuron weights are iteratively updated as follows:


(3) $$w^{n+1}=w^{n}+(-l\times \frac{\partial E}{\partial w}),$$

where *l* is the learning rate, $\frac{\partial E}{\partial w}$ is the error gradient with respect to the weights. The gradient shows how the function changes depending on the output variable. The feedforward multilayer perceptron is a universal approximator. After the signal is passed through the activation function, the output signal which *O* is the output signal of the neuron and is calculated as follows:


(4) $$O=f\left(\sum_{i}^{n}v_{i}w_{i}+p\right).$$

Neurons are grouped into layers, which are divided into input, output and hidden. The input layer receives the data coming to the input of the neural network and passes it to the next layer. The output layer has the same number of neurons as the number of classes in the data set, processes the information received from the previous layer and determines the output of the network. Hidden layers process the data received from the previous layer and calculate the output that is fed to the next layer.

As an activation function for the hidden layers of the proposed neural network system, the Rectified Linear Unit or ReLU is used, which is calculated as follows:


(5) f(s)=max{0,s}.

For the probabilistic distribution of input data over possible recognition classes, the *softmax* function is used on the output layer, which is calculated as follows:


(6) $$\sigma {\left(s\right)}_{j}=P(j|s,\theta )=softmax(s;\theta )=\frac{\exp(s_{j})}{\sum_{k=1}^{K}\exp(s_{k})}.$$

**FIGURE 4:**
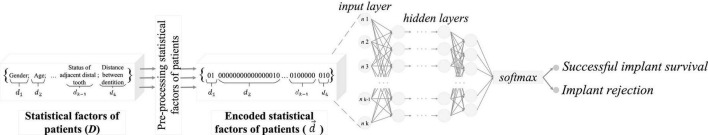
Architecture of the neural network system for predicting the survival rate of single implants.

The architecture of the proposed neural network system for predicting the survival rate of single implants is shown in Figure 4.

## Results

For the simulation, clinical cases of the experimental database of implant survival were used based on the description of the patient’s case histories. The database was collected using a multicenter retrospective and prospective study from patient records obtained according to a single protocol from dental implantation centers in such Russian cities as Stavropol, Moscow, Penza, Vladivostok, Grozny, Pyatigorsk, and Vologda. The collected and digitized database of implant treatment cases used for practical modeling is presented in LLC (2022). The collected and digitized database included 1,626 cases of dental implantation, which included 1,490 cases of successful implantation (91.64%) and 136 cases of implant rejection (8.36%). The dental database included digitized cases of implant treatment from 1998 to 2021. The average time of implant rejection was about 3 years. In most cases (87 patients out of 136), rejection occurred within the first year after implantation. The resulting base included 112 patient factors that affect the success of single implant survival, which could take 916 different values. Due to the fact that the collected database included 1,646 patient histories, training on a full experimental base is impossible due to the insufficient number of variations of all possible values of factors influencing implantation. Based on the recommendations received from specialists in the field of dental implantology, 55 factors were selected for modeling, divided into three groups (general somatic factors; the state of the dentoalveolar system; the state of the receptive bed). The factors used for neural network modeling and their cardinality are presented in Table 1.

**TABLE 1:** Table of cardinality of each factor selected for neural network modeling from the digitized database of cases of implant treatment.

No	Feature	Cardinality	No	Feature	Cardinality
**General somatic factors**			28.	Oral hygiene (Silness-Loe index)	9
1.	Gender	2	29.	Oral mucosal diseases	3
2.	Age	58	30.	Temporomandibular disorder	3
3.	Availability for constant monitoring	2	31.	Bruxism	2
4.	Features of professional activity	4	32.	Periodontal diseases	5
5.	Cardiac ischemia	5	33.	Condition of the right maxillary sinus	3
6.	Degrees of arterial hypertension	5	34.	Condition of the left maxillary sinus	3
7.	Drugs associated with diseases of the cardiovascular system	11	35.	Orthodontic treatment	3
8.	Kidney disease	3	36.	Dental implant treatment	4
9.	Diseases of the gastrointestinal system	6	**State of the receptive bed**		
10.	Endocrine disorders	3	37.	Implantation area (dental formula code)	32
11.	Diabetes mellitus	5	38.	Prostheses in the implantation area before surgery	3
12.	Diabetes mellitus medication	3	39.	Smile line	3
13.	Musculoskeletal disorders	2	40.	Tooth crown shape	2
14.	Previous implant treatment of a different nature	2	41.	Gingival biotype	3
15.	Complications of implant treatment	2	42.	Cause of tooth loss	4
16.	Osteoporosis	3	43.	Alveolar ridge protrusion level	6
17.	Maxillofacial oncology	5	44.	Defect form	4
18.	Oncological diseases of distant organs	3	45.	Bone density (quality)	5
19.	Bisphosphonate medication	2	46.	Bone level of adjacent teeth	3
20.	Anticoagulant medication	5	47.	Toothless gap width	3
21.	Hepatitis	4	48.	Bone grafting before implantation	4
22.	COVID-19	5	49.	Soft tissue grafting before implantation	2
23.	COVID-19 vaccination	2	50.	Sinus lift	3
24.	Mental disorders, according to the patient	2	51.	Adjacent medial tooth status	7
25.	Allergic reactions	4	52.	Adjacent distal tooth status	7
26.	Smoking	3	53.	Distance between dentitions	2
**State of the dentoalveolar system**			54.	Bone width (mm)	77
27.	Nutrition type	2	55.	Bone height (mm)	75
**Total**				**426**	

**FIGURE 5:**
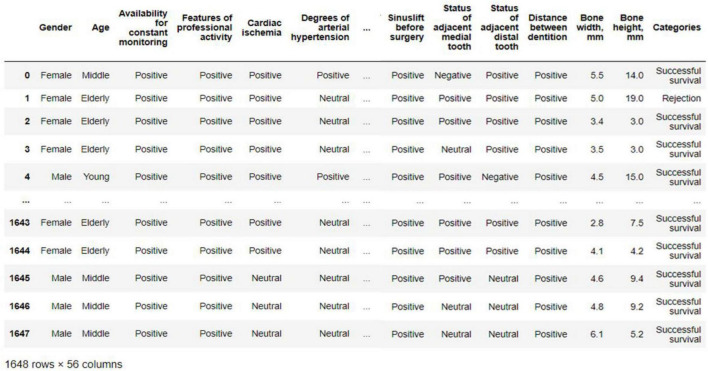
An example of the transformation of variables of the experimental implant survival database based on the description of patient histories.

The parameter of implant survival is represented by two possible values—“Successful survival,” “Rejection,” which are classes for neural network recognition of clinical cases. The parameter “Age at the time of implantation, years” was divided into three groups in accordance with the age classification adopted by the World Health Organization (WHO). The first group of “young age” is represented by patients under the age of 44 years. The second group of “middle age” is represented by patients aged 45–59 years. The third group of “elderly” is represented by patients aged 60 years and above. Also, in order to reduce the variability of the selected parameters, based on the recommendations, the available data were processed. Each factor was converted to one of three possible values - a positive effect (the variable value is “positive”), a neutral effect (the variable value is “neutral”), or a negative effect on implant success (the variable value is “negative”). Thus, it was possible to reduce the number of possible values that the selected 55 factors can take from 426 to 164. Table 2 presents the factors selected for neural network modeling and their cardinality after pre-treatment to reduce variance. An example of transformed variables is shown in Figure 5.

**TABLE 2:** Table of cardinality of each factor selected for neural network modeling after pre-processing to reduce variability.

No	Feature	Cardinality	No	Feature	Cardinality
**General somatic factors**			28.	Oral hygiene (Silness-Loe index)	3
1.	Gender	2	29.	Oral mucosal diseases	3
2.	Age	4	30.	Temporomandibular disorder	2
3.	Availability for constant monitoring	2	31.	Bruxism	2
4.	Features of professional activity	3	32.	Periodontal diseases	3
5.	Cardiac ischemia	3	33.	Condition of the right maxillary sinus	2
6.	Degrees of arterial hypertension	3	34.	Condition of the left maxillary sinus	2
7.	Drugs associated with diseases of the cardiovascular system	1	35.	Orthodontic treatment	3
8.	Kidney disease	3	36.	Dental implant treatment	3
9.	Diseases of the gastrointestinal system	3	**State of the receptive bed**		
10.	Endocrine disorders	3	37.	Implantation area (dental formula code)	3
11.	Diabetes mellitus	3	38.	Prostheses in the implantation area before surgery	3
12.	Diabetes mellitus medication	1	39.	Smile line	3
13.	Musculoskeletal disorders	2	40.	Tooth crown shape	2
14.	Previous implant treatment of a different nature	1	41.	Gingival biotype	3
15.	Complications of implant treatment	1	42.	Cause of tooth loss	3
16.	Osteoporosis	3	43.	Alveolar ridge protrusion level	3
17.	Maxillofacial oncology	3	44.	Defect form	3
18.	Oncological diseases of distant organs	3	45.	Bone density (quality)	3
19.	Bisphosphonate medication	1	46.	Bone level of adjacent teeth	3
20.	Anticoagulant medication	2	47.	Toothless gap width	3
21.	Hepatitis	2	48.	Bone grafting before implantation	2
22.	COVID-19	2	49.	Soft tissue grafting before implantation	2
23.	COVID-19 vaccination	1	50.	Sinus lift	3
24.	Mental disorders, according to the patient	2	51.	Adjacent medial tooth status	3
25.	Allergic reactions	3	52.	Adjacent distal tooth status	3
26.	Smoking	3	53.	Distance between dentitions	2
**State of the dentoalveolar system**			54.	Bone width (mm)	19
27.	Nutrition type	1	55.	Bone height (mm)	14
**Total**				**164**	

For modeling, 1,626 clinical cases were used, which were divided into data for training and testing in a percentage ratio of 80–20. The simulation was carried out using the high-level programming language Python 3.8.8. The Pytorch machine learning framework was used to model the neural network system. The NumPy, Pandas, and Scikit Learn libraries were used to process statistical data. The Matplotlib library was used to visualize the data. Each neural network system was trained for 100 epochs. When using a larger number of epochs, a pronounced retraining of each of the proposed neural network systems was observed. The batch size was 16. Adam was used as an optimizer with a standard learning rate of 0.001. CrossEntropyLoss function was used as an error function. All calculations were performed on a PC with an Intel^®^ Core™ i5-8500 processor at 3.00 GHz with 16 GB of RAM and a 64-bit Windows 10 operating system.

**FIGURE 6:**
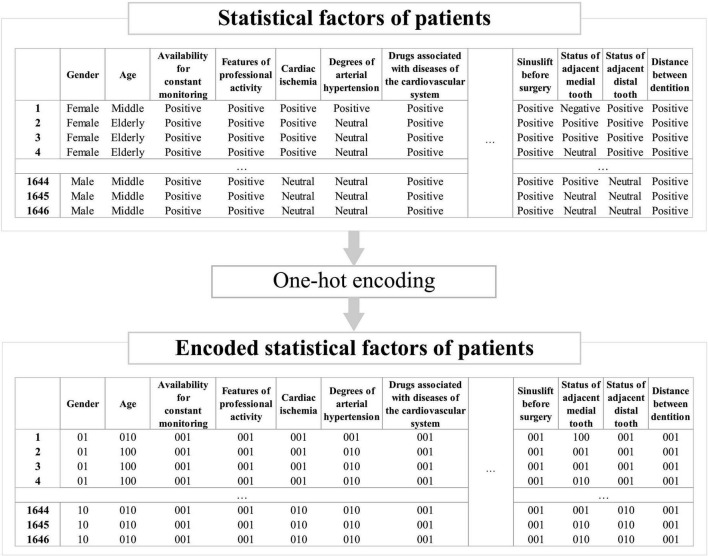
An example of pre-processing patient factors using the one-hot encoding method.

The simulation data was pre-processed using the one-hot encoding method in order to convert it into the vector format required for further analysis. Coding tables for each possible value of patient factors affecting implant survival are presented in Tables 3–5. An example of patient factor pre-processing using the one-hot encoding method is shown in Figure 6.

**TABLE 3:** Coding table using the one-hot encoding method for the statistical factor of the patient’s gender.

Patient gender	One-hot code	
Female	0	1
Male	1	0

**TABLE 4:** Coding table using the one-hot encoding method for the statistical factor of the patient’s age.

Patient age	One-hot code		
Young	0	0	1
Middle	0	1	0
Elderly	1	0	0

**TABLE 5:** Coding table of patient’s statistical factors using the one-hot encoding method.

Patient age	One-hot code		
Positive	0	0	1
Neutral	0	1	0
Negative	1	0	0

The main difficulty in choosing the optimal neural network architecture using evolutionary algorithms (Hamdia et al., 2021) is that they are time-consuming, computationally intensive, and demanding on user-defined parameters (Abbasi et al., 2015). In contrast, non-evolutionary algorithms and, in particular, trial-and-error method (Sun et al., 2008) require much less time for simulation and user parameters. Thus, in order to effectively select the optimal neural network topology for the system for predicting the risk of complications in implant treatment of the maxillofacial region, a number of experimental simulations of various neural network architectures were carried out using the trial-and-error method.

For the task of assessing the risk of complications in the implant treatment of pathologies of the maxillofacial region, the data for modeling include an expert assessment of the success of implant survival. Thus, a multilayer perceptron with direct connection was chosen as the type of neural network. The activation function used was the ReLU function, which is the most commonly used activation function in deep learning. The ReLU function has such advantages over the sigmoid and hyperbolic tangent as a quick and easy calculation of the derivative, as well as activation sparseness, which allows to reduce the number of neurons to turn on (Li and Yuan, 2017).

**FIGURE 7:**
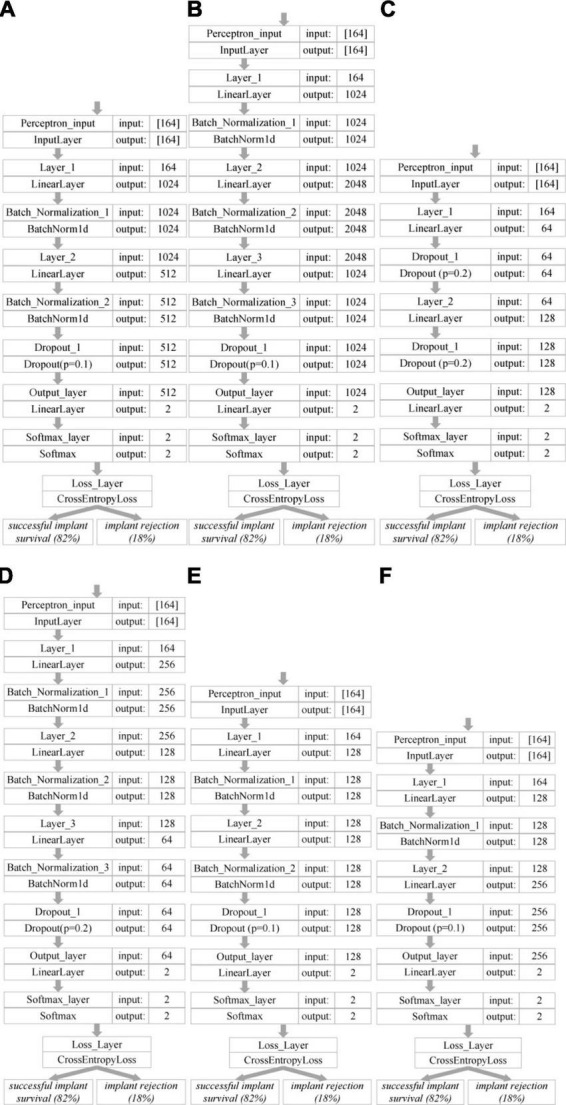
Developed architectures of neural networks for predicting the success of survival of single implants: **(A)** No. 1; **(B)** No. 2; **(C)** No. 3; **(D)** No. 4; **(E)** No. 5; **(F)** No. 6.

Various linear neural network architectures were used for modeling. The input of each neural network was a data vector of 164 characters after preprocessing by the one-hot encoding method of each clinical case from the database. As a result of a number of experimental simulations, 6 variants of the neural network topology were developed to predict the survival rate of single implants in the surgical treatment of pathologies of the maxillofacial region, which made it possible to obtain high results in the accuracy of recognizing the success of implant survival. The developed architectures of neural networks for predicting the success of implant survival are shown in Figure 7. The development of neural network architectures was carried out by trial and error. From the developed and trained neural network architectures, six most successful topologies were selected, which made it possible to obtain the best result in prediction accuracy. At the same time, in each of the neural network architectures, the number of neurons and the number of layers changed. It was found that a gradual decrease in the number of neurons makes it possible to obtain the highest results in the accuracy of predicting the survival rate of single implants.

For neural network architectures No. 1 and No. 4, a gradual decrease in the number of neurons on each new layer was carried out. For neural network architecture No. 2, the number of neurons was increased by two times and then the number of neurons was reduced by two times. For neural network architecture No. 3, a reduction and subsequent increase in the number of neurons was made. For neural network architecture No. 5, the number of neurons was constant and did not change throughout the layers. For neural network architecture No. 6, a slight reduction in the number of neurons was made and a further increase by two times.

Table 6 presents the results of evaluating the developed neural network systems for predicting the survival rate of single implants in the surgical treatment of pathologies of the maxillofacial region. The highest accuracy in predicting the survival rate of single implants was achieved using neural network architecture No. 4 and amounted to 94.48%. An increase in the number of neurons in the first layer and their gradual decrease in the subsequent ones in neural network architectures No. 1 and No. 4 made it possible to achieve the highest accuracy in predicting the success of implant survival. With an increase in the number of layers in neural network architectures, there was an increase in the time spent on training without a significant increase in the prediction accuracy index of the neural network. It was found that for the task of classifying statistical data of patients, the architecture consisting of four linear layers with a gradual decrease in the number of neurons is optimal in terms of accuracy and time costs.

**TABLE 6:** Results of modeling the developed architectures of neural networks for predicting the survival rate of single implants.

CNN architecture	Loss function	Accuracy, %	F1-score	Recall	Specificity	Time, s.
No. 1	0.2529	94.17	0.9657	0.9837	0.9565	98.77
No. 2	0.2914	93.86	0.9386	0.9386	0.9497	508.33
No. 3	0.6931	93.87	0.9386	0.9386	0.9386	57.97
No. 4	0.2366	94.48	0.9657	0.9837	0.9565	52.36
No. 5	0.2455	93.25	0.9141	0.9542	0.9542	43.14
No. 6	0.2770	92.94	0.9295	0.9294	0.9492	34.62

The F1-score corresponds to the harmonic mean of the accuracy and recall of the neural network model and is used as one of the estimates of binary classification systems. The highest F1-score was obtained for high-precision neural network architectures No. 1 and No. 4 and amounted to 0.9657. The lowest F1-score was obtained when testing the neural network architecture No. 5, the distinctive feature of which is the constant number of neurons on each neural layer.

Recall is used as a statistical measure in cases where the cost of false negatives is high. In the field of medicine, and in particular, dental implantology, neural network false negative prediction is more dangerous due to potential risks to the health of patients. The best recall rate when testing the developed systems for predicting the survival of single implants was obtained with neural network architectures No. 1 and No. 4 and amounted to 0.9837.

Specificity is a statistical metric that is defined as the proportion of true negative neural network prediction results. For neural network systems in the field of medicine and, in particular, dental implantology, when the data are highly unbalanced toward positive cases (successful survival of implants), the specificity indicator is an important statistical measure of model evaluation. The highest specificity index was obtained for neural network models No. 1 and No. 4 and amounted to 0.9565.

## Discussion

As a result of the study, a neural network system was developed for predicting the success of single dental implants with a test accuracy of 94.48%. The developed neural network system analyzes 55 statistical factors of patients, which are the general somatic factors of the patient, the state of the dentoalveolar system, the state of the perceiving bed. The collection and digitization of a large number of case histories made it possible to create a unique training database for training artificial intelligence. Due to the multivariate analysis of statistical data of patients, the developed neural network system makes it possible to predict the success of single implants with high accuracy. Table 7 compares the accuracy of predicting the survival rate of single implants in similar neural network systems with the proposed neural network system.

**TABLE 7:** Results of testing various systems based on artificial intelligence to predict the success rate of dental implants.

Neural network system for analyzing the statistical factors of patients for predicting the survival rate of dental implants		The accuracy of predicting the survival of dental implants, %
Known neural network systems	Sadighpour et al., 2014	83.30
	Braga et al., 2012	78.90
	Oliveira et al., 2005	75.90
	Liu et al., 2018	74.10
The proposed neural network system for predicting the survival rate of dental implants		94.48

The article Sadighpour et al. (2014) presents an AI-based system with a forward/backpropagation architecture for determining a plan for the surgical placement of dental implants. Modeling was carried out on digitized 47 clinical cases of osseointegration. Each clinical case included 9 possible variables. Recognition was made according to two possible categories, such as a general treatment plan or a detailed treatment plan. In the case of a detailed treatment plan, there was a high risk of dental implant rejection. The proposed neural network architecture included 9 input neurons, a hidden layer with 20 neurons, and an output layer with 2 neurons. The accuracy of the proposed neural network system from the study Sadighpour et al. (2014) was 83.30%, which is 11.18 percentage points lower than the proposed system for predicting the success of single implants. A significant difference in the accuracy of the considered systems may be due to the amount of training data as well as the optimality of the chosen neural network architecture. Modeling on an insufficient amount of data with a large number of various statistical factors affecting osseointegration can lead to unreliable obtaining of weight coefficients during training. At the same time, the number of possible combinations for 9 statistical factors is significantly higher than the amount of data used for training and testing. The absence of recurring combinations of statistical factors makes it impossible to determine the pattern between the possible rejection of a dental implant and the statistical information available in a particular case that affects osseointegration.

The study Braga et al. (2012) presents a binary neural network model for predicting the success of dental implant survival. Modeling was carried out on the basis of a database of 155 cases of implantation of patients. Moreover, each case included 57 variables that determine the possible risks of implant treatment. In addition to general somatic factors, as well as factors of the state of the dentition, data related to the genetic characteristics of patients, such as the presence of cytosine, thymine in local genes, etc., were used. As a result of testing five different neural network models, the highest accuracy result on the test set was 78.90% for neural network model No. 4. The results obtained are 15.58 percentage points lower than the test accuracy of the proposed neural network system for analyzing patient statistical factors for predicting the success of single dental implants. The low recognition accuracy of the considered model from Braga et al. (2012) can be explained by the insufficient number of training examples for 57 factors affecting osseointegration. With such insufficient data for modeling, it is almost impossible to repeat the combination of the 57 factors considered to find a pattern between the patient’s condition and implantation success.

The paper Oliveira et al. (2005) presents a comparative study of four machine learning methods for predicting the success rate of dental implants. Algorithms based on neural networks, support vector machines and K-nearest neighbors have been proposed. The modeling data set consisted of 157 patient statistics examples. Each training example included 7 statistical factors such as age, gender, implant position, implant type, surgical technique, smoking, and pre-existing medical conditions. All patient factors could take 17 possible values. At the same time, the classification of the analyzed data was carried out according to 7 possible categories. As a result of the simulation, the highest accuracy in predicting the successful survival of dental implants using the neural network algorithm was 75.90%, which is 18.58 percentage points lower than that of the proposed system based on artificial intelligence. A significant difference in prediction accuracy can be explained by a multivariate analysis of the statistical data of patients, as well as by training the proposed system on a large amount of digitized data with a more complete description of the factors affecting osseointegration.

In Liu et al. (2018) a model for predicting and early warning of potential implant rejection was developed. Modeling was carried out on the basis of clinical cases of 681 patients. Data were collected for each patient, including 20 factor variables. As a result of modeling using supervised learning methods, a neural network system was obtained with a prediction accuracy of 74.10%, which is 20.38 percentage points lower than the proposed neural network system for predicting the success of single dental implants. The difference in the results of the test prediction accuracy can be explained by the insufficient number of analyzed factors affecting osseointegration, as well as the insufficient amount of data for modeling.

The main limitation of using the proposed neural network system for analyzing patient statistical factors to predict the success of single dental implants is that dentists and specialists can only use the system as an additional diagnostic tool. The proposed system is not a medical device or program and cannot self-diagnose patients. At this stage, the developed neural network system is capable of predicting a positive or negative outcome of a single dental implant operation and cannot be used as a full-fledged tool for supporting medical decision-making. Since the majority of implant treatment cases describe successful engraftment of the implant during osseointegration, cases of false negative classification are possible.

## Conclusion

The paper presents an artificial intelligence-based system for analyzing the statistical factors of patients in order to predict the success of dental implant survival. The collected and digitized database of clinical cases of osseointegration, as well as the neural network architecture optimally designed for the collected factors, made it possible to obtain a neural network system with a test accuracy of 94.48%. The proposed system based on artificial intelligence makes it possible to achieve higher prediction accuracy than similar neural network systems due to the analysis of a large number of statistical factors of patients, as well as deeper learning on a large amount of data. The use of the proposed system based on artificial intelligence as an additional auxiliary tool will allow the dentist to pay attention to minor factors that affect the quality of the installation and further survival of the implant, and reduce the percentage of complications at all stages of treatment.

The main limitation of using the proposed neural network system for predicting the survival rate of single dental implants is that specialists can use the system only as an additional diagnostic tool. The proposed system cannot be used as a full-fledged tool for supporting medical decision-making.

A promising direction for further research is the development of a medical decision support system based on the technology for generating recommendations to reduce the risk of complications, indicating certain factors that affect the clinical situation. It is also planned to develop of methods for minimizing false negative classification through the use of weighting factors. In further studies, it is planned to develop a detailed neural network study module to obtain the percentage of influence of each factor on the overall picture of the clinical case. Further development of a medical decision support system based on the proposed neural network system will make it possible to determine specific recommendations for the doctor and patient in order to identify the most potentially dangerous factors that negatively affect osseointegration to further minimize complications at all stages of implant treatment.

## Data availability statement

The raw data supporting the conclusions of this article will be made available by the authors, without undue reservation.

## Author contributions

All authors listed have made a substantial, direct, and intellectual contribution to the work, and approved it for publication.
